# Preparation for teacher collaboration in inclusive classrooms – stress reduction for special education students via acceptance and commitment training: A controlled study

**DOI:** 10.1186/s40303-015-0015-3

**Published:** 2015-09-28

**Authors:** Simone Pülschen, Dietrich Pülschen

**Affiliations:** Institute of Special Education, Department of Special Educational Psychology, Europa-Universität Flensburg, Auf dem Campus 1, 24943 Flensburg, Germany; Department of Psychiatry, University of Rostock, Gehlsheimerstr. 20, 18147 Rostock, Germany

**Keywords:** Cognitive behavioral therapy, Acceptance and Commitment Therapy, Collaboration, Stress reduction, Evidence-based practices, Intervention strategies, Teaming, Health promotion

## Abstract

**Background:**

The education system in Germany is beginning to witness a sea change, lately, owing to the country’s ratification of the United Nation’s Convention on the Rights of Persons with Disabilities. The enactment is aiming at making provision for special education teachers to share the same teaching platform and institution with other teachers for teaching children from all backgrounds, irrespective of their needs. While promoting the benefits of collaborative teaching, this provision would also effectively establish role demarcation among teachers. However, the level of participation and adaptiveness displayed by individual teachers would play a major role in determining the success or failure of the intended collaborative framework.

Collaboration also becomes challenging due to the level of stress involved in the teaching profession. The fact that only 65 % of teachers in Germany reach retirement age while still in service, primarily due to psychiatric illness, has posed questions on adopting the collaborative framework for teachers from diverse backgrounds. In other words, it can be stated that the process of collaborating with teachers from different professional backgrounds and with varying levels of skills will potentially lead to further stress. The stress-related psychological states, developed through the collaborative processes, might affect the biological stress-response systems of the participating teachers. With stress-response contributing directly to the pathogenesis of stress-related diseases and disorders in the long term, it would be important to contain the ripple effect of collaborative framework that the enactment intends to establish between SEN (special educational needs) teachers and others.

**Methods:**

In addition to impacting the long-term health of teachers, the collaborative framework is also suggestive of having similar effects on students studying special education (SEN students). A study was conducted to examine the stress levels associated with the collaborative framework. An expression in terms of two (group affiliation) × 2 (measurement time) between subjects design was implemented to examine the effects of an Acceptance and Commitment Training on the subjective tension of a sample (*N* = 68) of SEN students. The sample was split into an intervention and a control group (IG and CG). The effects of the training on collaborative competence were examined using the Chi-square test. Questionnaire and role plays were used to assess the collaborative competence and the subjective tension.

**Results:**

The participants had significant stress levels and displayed an uncooperative attitude during the initial assessment. However, these results reversed after the Acceptance and Commitment Training. Significant decrease in stress levels and improved cooperation were evident among the participants in the intervention group, as opposed to the participants of the control group.

**Conclusions:**

The findings of this study show that the Acceptance and Commitment Training is an appropriate medium to establish and develop collaboration skills, and an effective technique to reduce high levels of subjective stress. Furthermore, the training evaluation and feedback indicate that it is well-accepted by all participants. The training is also endorsed as a practically relevant medium to help SEN students collaborate and combat stress.

## Background

Researches related to stress show that the brain, being the central organ for stress response, determines the elements or factors of stress [1]. The selection of a method to deal with stress would depend on whether or not a situation is considered as stressful. Therefore, it is important to identify the factors that contribute to stress. This study will focus on factors responsible for stress in school environment, especially teachers’ stress levels. Teaching is a profession linked to high levels of stress. An increasing number of studies point towards the stress levels in school workforce, while listing a number of stress triggers in the working environment at school. The possible stress triggers identified in these studies include high work load, noise, communication hurdles with pupils, parents, other teachers or the principal [2]. Vague or aversive job profiles, wide ranging and highly complex tasks as well as role conflicts also characterize stressful situations [3].

Studies show that only 65 % of German teachers reach the legal pension age before they retire [4], while most of the teachers retire early due to psychiatric illness [5]. In other words, psychiatric instability has been responsible for early retirement for a significant percentage of teachers in Germany. This scenario has worsened since Germany’s ratification of the UN Convention on the Rights of Persons with Disabilities in 2009. Although the enactment is transforming the educational system in Germany, it is also becoming responsible for creating stressful environment for the teachers.

Special education schools are on the verge of closure (at least in the state of Schleswig-Holstein), and there are plans to bring special educators and all other teachers to a common teaching platform. The SEN teachers and other teachers would be catering to all children, irrespective of their needs, under the same roof. In addition, these teachers would be expected to assume the roles and responsibilities of a “team player”, under the new scheme of things. While a window of opportunity has opened for all teachers, lack of clarity with respect to roles, objectives and task sharing might pose as an obstacle to teachers in their way of realizing this opportunity. While there are no doubts regarding the importance of collaboration among teachers to facilitate successful integration [6, 7], several research and field reports have illustrated the complexities involved in establishing effective collaborative practices. The teachers in Germany are compelled to support government decisions [8], by virtue of being public servants. The teachers might express similar reluctance in response to the government’s new agenda, which would involve role change and creation of a collaborative teaching environment.

The statutory framework for the UN Convention in Germany is provided by the “Standing Conference of the Ministers of Education and Cultural Affairs of the Federal States (Länder) in the Federal Republic of Germany” (abbr.: Standing Conference), within their recommendations on inclusive education of children and young people with disabilities in schools (“Inklusive Bildung von Kindern und Jugendlichen mit Behinderung in Schulen”) [9]. According to the Standing Conference, it is the responsibility of the Federal States to adapt teacher training to new challenges and to provide recommendations for teacher collaboration in inclusive settings. The current scenario does not provide any framework for organizing collaborative working practices among teachers, and individual teachers are expected to develop their own methods to achieve collaboration in the work environment. This study aims to formulate methods for improving collaboration between teachers in inclusive classrooms, and simultaneously identify means to reduce stress from their scope of work.

The current study deals with four different aspects given below:

- Teacher professionalization, especially in the context of inclusive classrooms
- Stress
- Psychotherapy
- Conflict management.

The above aspects are elaborated below and the subsequent hypotheses are also verbalized.

### Competence-oriented teacher professionalization

The framework of this study is based on the recent government agenda of teacher professionalization. The job specifications for teachers are well-documented on the national and international level, and the agreed standards for competence-oriented teacher professionalization include knowledge, skills, and commitment. These are considered to be primary requisites for candidates aspiring to become teachers in Germany [10]. However, this study is focusing on commitment and committed actions, which are instrumental in building collaborative competence.

While taking a systemic approach, Heimlich expressed that pedagogical acting in inclusive classrooms is always collaborative acting, which is also based on personal, social, and professional skills [11]. The definition of collaboration as given by Friend and Cook, they state, “Interpersonal collaboration is a style for direct interaction between at least two coequal parties, voluntarily engaged in shared decision making as they work toward a common goal” [12]. Furthermore, personal commitment and communication skills, especially in interactions related to problem solving or conflict management, are necessary for collaboration in inclusive classrooms. Therefore, the present study refers to this competence as collaborative competence. It is necessary to provide training on collaborative competence as early as possible during teacher preparation programs. However, it is critical for teachers to engage in self-reflection and be open to receiving feedback for achieving collaborative competence. While feedback promotes skill development and competency, self-reflection enables teachers to identify areas of strengths and improvement required for personal growth [13].

### Stress in inclusive classrooms

According to Hillert, Koch and Lehr, 65 % of German teachers claim the state pension due to early retirement [1]. Numerous studies confirm that many teachers in Germany opt for an early retirement, and psychiatric diagnoses are associated with early retirement of teachers [14]. The development of this kind of disorder is often related to the malfunction of the stress system [4, 15].

The key elements of stress have been divided into three distinct categories, based on stress-related research that is conducted over the years. Environmental, psychological and biological phenomena are considered as decisive factors in the field of stress, and these factors have led to the emergence of distinct views on matters related to stress [16]. It is observed that the imbalance between environmental demands and a person’s adaptive capacity often triggers a biological and psychological response. Concerning the stress response, a distinction must be drawn between the behavioral and physiological consequences of an individual’s response to (chronic) stressors.

Within the context of the biological stress response, it is the hypothalamic-pituitary-adrenal (HPA) axis along with its end-product cortisol that regulates the long-term adaption to stress (work-stress included) [17]. If a situation is interpreted as being stressful, then it leads to the activation of the HPA axis and the secretion of cortisol and catecholamines. These physical processes are referred to as fight-or-flight-response. As described in this definition, “the fight-or-flight-response circumscribes the allocation of physiological energy in order to facilitate fighting the imminent danger or escaping from threat” [18]. This physical activation is designated as ‘allostasis,’ a term introduced by Sterling and Eyer [19]. Considering it from an evolutionary biology point of view, allostasis is a necessary process to ensure survival. However, when an individual is unable to adapt to the behavioral fight or flight response and hence fails to achieve allostasis, there is a build-up of allostatic load. This pressure on the body’s stress response system is also termed as allostatic overload [4]. As components of the biological stress response, automatic changes in the central nervous system have been identified for causing alterations in cardiovascular, immunological and other physiological systems. It is a known fact that the dysregulation of these systems is responsible for a wide variety of physical health problems [16]. Although several studies agree that a general dysregulation of the HPA axis is involved in the pathogenesis of stress-related illnesses, the results regarding the direction of this dysregulation are contradictory. Multiple studies confirm that the hyper-(re)activity as well as the hypo-(re)activity of the axis make it difficult to interpret results from the existing cortisol literature, if available [20]. Additionally, at the moment, very little psychoneuroendocrinological research has been conducted in the field of stress-related illnesses among teachers. Stress-related research concerning teachers primarily focuses on behavioral stress mechanisms. According to Contrada, it is a cognitive perspective that dominates research concerning the initiation of stress in general [16]. The interpretation or perception of stress differs from person to person, and is determined by their ability to cope with a stressful situation. In addition, cognitive behavioral interventions, which are formulated to improve an individual’s coping ability, can produce positive effects on the psychological and behavioral outcomes of the concerned individual—the behavioral stress response refers to a direct and quick activation of negative emotions and burdening thoughts in a stressful situation [5]. Contrary to the biological stress response, the behavioral stress response is consciously perceived and related to certain emotional states like a feeling of fear, anger, helplessness or guilt, and so on. There is also a cognitive component, which is characterized by increased mental occupation and burdening thoughts. Referred to as aversive cognitions, these thoughts include melancholy, fright, self-criticism, etc. An individual’s coping capability could be improved by changing the way of dealing with aversive cognitions.

While there are cognitive interventions to improve an individual’s coping ability, external measures are required to avoid scenarios that cause stress. With regards to government’s agenda to facilitate collaborative teaching, measures to create conducive conditions for collaboration in inclusive classrooms are non-existent. Considering individual struggles involved in the collaborative process, absence of desirable environment for collaboration could trigger a stress response in the workplace. Facts suggest that collaboration involves multiple, and complex tasks, and therefore the collaborative process [6, 7, 21, 22] is considered to be a potential stressor. Besides the stress involved in the collaborative process, as mentioned above, vague job profiles, wide-ranging and highly complex tasks, and role conflicts have been adding to the stress levels of the teachers [5].

### Psychotherapy methods in teacher trainings

As stated above, the behavioral stress response is related to increased mental occupation and burdensome thoughts. Currently, the clinical-psychotherapeutic arena is witnessing state-of-the-art psychotherapeutic techniques, which is termed as ‘third wave psychotherapies’ or ‘third wave’ cognitive and behavioral therapies (CBT) [23]. These psychotherapeutic methods aim to treat aversive cognitions and promote psychological flexibility. Acceptance and Commitment Therapy (ACT, pronounced like the verb) is one of the ‘third wave’ CBTs [24]. When analyzed from an ACT perspective, transforming an individual’s relationship with burdensome and stressful thoughts is more effective and crucial than altering dysfunctional emotions and cognitions. The main goal of the ‘third wave’ CBT is to enable an individual to realize that acceptance of stressful thoughts, guided by personal values, can produce positive results during the treatment of any stress-related disorder. This attempt of the latest psychotherapeutic technique effectively develops psychological flexibility [25]. Thus, it can be stated that value clarification is one of the priorities of ACT. Within the regimen of ACT, effective action can be encouraged by setting goals in accordance with personal values. This goal-setting can facilitate attitude alteration, and hence improve an individual’s capability to cope with stressful situations.

In the 1970s and 1980s, it was a familiar practice to include psychotherapeutic methods in the teacher training programs [26]. The following study remains true to this tradition and integrates ACT into teacher training for promoting collaborative competence. The aim of this integration in the collaborative teaching framework is to reduce stress in inclusive settings, which involve special education students.

With an aim to train teachers for this setting, the ACT implementation can fall back on research of Bond and colleagues [27–30], who implemented ACT at work to reduce stress. Flaxman and Bond pointed out that their protocol might be used in “training programs for other population that have to cope with demanding situations”. Based on this finding, it can be stated that ACT can be implemented for teachers in inclusive classrooms [31]. As portrayed through the research of Bond and Bunce, the changes in habits, as promoted by ACT, can have a positive influence on stress-reduction in the target setting [28]. Participants of the ACT training showed significantly reduced stress levels, improved psychological well-being, and a higher propensity to innovate. This finding reveals that ACT can be more effective in a prevention setting than in a therapeutic setting, and therefore the use of the term training in the expanded form of ACT (Acceptance and Commitment Training) is justified in this study.

Under the purview of the Commitment training section of ACT, additional Nonviolent Communication (NVC) training [32] will be provided to the teachers. The practice of NVC will help future teachers to express observations without attaching any bias, personal sentiments or judgmental perspectives. Teachers will be able to contribute to each other compassionately, and resolve conflicts effectively, peacefully, and adaptively. The main goal of NVC is to improve the quality of personal and professional relationships through effective communications. Until now, no research has been conducted that would integrate NVC with ACT. Although there are several limitations affecting the outcome evaluation, findings from the current research on NVC point out that NVC trainings deliver promising results [33–35].

### Conflict management

Along with the definition of a collaborative competence, the ability of students to solve conflicts was addressed during the study. The study also emphasizes the importance of conflict management. Research in the field of conflict handling, according to Blake and Mouton, often focuses on the five conflict handling modes [36]. As per the management theorists, the conflict handling modes differ by two basic dimensions of conflict behavior. The first dimension is referred to as assertiveness, which is defined as a person’s attempt to satisfy his or her own concerns. The second dimension of cooperativeness is defined as an individual’s attempts to satisfy the concerns of another person [36].

The theory of conflict management is expounded by Schwarz [37]. He develops an ascending order for the five modes for developmental considerations. In addition, to accommodate the accompanying learning process in every conflict, Schwarz adds a sixth conflict handling mode. These modes are as follows:

1. Avoiding (flight): an individual does not address the conflict.
2. Competing (destruction): an individual pursues only his or her own concerns.
3. Accommodating (subordination): an individual pursues only the concerns of the other person.
4. Delegating: Schwarz integrates the delegation of a conflict in his conflict theory. He emphasizes on the existence of a right and a wrong solution for a third-party conflict resolution. The third party should take on the role as a mediator.
5. Compromising: in the middle of competing and accommodating, wherein an individual tries to find a solution, which partially satisfies both parties. Schwarz describes ‘compromising’ as the preliminary stage of ‘collaborating’.
6. Collaborating: an effort to meet both sets of concern, wherein an individual tries to find a solution, which fully satisfies both parties (exploring the disagreement is necessary for this conflict handling mode). Collaborating is the highest level in conflict solving because it helps establish a lasting relationship between the disputants. Collaboration also improves future communication between the disputing parties.

This hierarchy can also be seen as a ‘ladder of maturation’ with ‘avoidance’ at its bottom and ‘collaboration’ at its top.

The aim of the present study is to evaluate and develop an education module. Through the ACT implementation, the module would prepare special education students for communication, interaction and problem solving in inclusive settings. As stated earlier, this study is based on the hypothesis that psychoeducational training (Acceptance and Commitment Training) linked with practical experience can increase collaborative competence. The outcome of this training would be as follows:

1. A reduction of subjective tension (H1) and
2. An effective and successful conflict handling mode − collaboration (H2).

Accordingly, subjective tension and the conflict handling mode represent the dependent variables in this study.

## Methods

A suitable measurement method for assessing successful communication, interaction and conflict handling (a collaborative competence) would be crucial for the development and evaluation of the aforementioned education module. The assessment of knowledge alone is not enough for carrying out processes such as communication, interaction or conflict handling. As stated in this parameter, “Performance assessments generally require the test takers to demonstrate their abilities or skills in settings that closely resemble real-life settings” [38]. The training discussed in this study implements the role-playing approach, which was also suggested to be an useful tool for teacher education by the Standing Conference [39].

### Design and procedure

This study used a quasi-intervention design with two measures for two groups − the intervention group (IG) and the control group (CG). The ACT for the IG took place during the course ‘Counseling, collaboration and conflict handling’, within the master program ‘Special Education’, which was held at the Europa-Universität Flensburg from April to July 2013, for 12 fixed dates. A total of 33 out of 40 students agreed to be voluntarily engaged in this study. The control group was enrolled to other seminars in the master program, which included basics of conversation and discussion management as well as role plays. A total of 35 out of 47 students voluntarily agreed to be part of the control group.

An assessment of the level of difficulty of different conflict situations was conducted by means of a questionnaire. This assessment was coupled with behavior observation, which was carried out in a setting that closely resembled real-life settings; here the setting was a role play between a teacher (taken over by an amateur actor—one of the authors of this article) and a special educator represented by the student. The combination of these evaluations represented an economical way to assess the development of the collaboration competence.

The subjects ranked 12 possible conflict situations—collected during a preliminary study—for conducting self-assessment in the area of collaboration. The behavior observation was standardized and the role plays were video recorded. The students ranked 12 situations (from 1 to 12) as part of the first step of the study. The ability to collaborate was demonstrated in a conflict situation with a mean level of difficulty. The situations ranked in the 6th position were identified in the mean level of difficulty and hence chosen for the role play. Subsequent to the role play, the subjective tension of each subject was identified by choosing a number on a scale from 0 to 100. It was also important to identify whether the tension emerged from personal disposition or practical contention. The amateur actor was briefed to act as a rigid and non-collaborative teacher. The role play determined different behaviors of the characters. The role plays were assessed on the basis of the conflict handling mode theory of Blake and Mouton and Schwarz [36, 37] with slight changes:

- Successful conflict solving only occurs when the concerns of disputing parties are met and satisfied—conflict-mode of collaboration. Compromising being a pre-step to collaborating, both conflict modes were combined in this mode, and named as collaborating.
- ‘Competing’ and ‘accommodating’ remain identical with those defined by Blake and Mouton [36] and Schwarz [37].
- The conflict mode ‘delegating’ was split into ‘delegating’—within the context of mediation—and ‘escalating’—within the meaning of escalating the conflict to a superior, the principal. Role plays, wherein the disputing parties are asked to seek a solution that meets both their concerns, fail to present a wrong or right solution.
- Avoiding was not integrated as part of the intervention because the subjects were forced to address the conflict.

With the measurement of the base level (T1), the subjects were asked to provide information about their practical experience in inclusive classrooms (internship or work experience) and their level of identification with the concept of inclusion (yes or no). The measurement of base level was followed by the ACT training for the IG, lasting for three months. The second phase of measurement of the dependent variables (T2) was carried out after the three-month period, along with questions about the acceptance and evaluation of the ACT.

### Participants

As already mentioned above, the sample was divided into two groups. The CG comprised 28.6 % male students and the IG was composed of 27.3 % male students (Table 1). In both groups, participants were special education students of the Europa-Universität Flensburg. The IG comprised only Masters students, as ACT was part of the compulsory course in the Masters curriculum. However, 68.4 % students from the Bachelors stream participated in the CG. The two groups did not differ with regards to age, as confirmed by *t*-test. The average age of the CG and IG were *M* = 25.9 (SD = 5.5) and *M* = 27.0 (SD = 2.3), respectively.

**Table 1:** Distribution of sex and semester

			CG	IG	Total
Sex	Female	N	25	24	49
		%	71.4 %	72.7 %	72.1 %
	Male	N	10	9	19
		%	28.6 %	27.3 %	27.9 %
	Total	N	35	33	68
		%	100.0 %	100.0 %	100.0 %
Semester	Bachelor	N	24	0	24
		%	68.6 %	0.0 %	35.3 %
	Master	N	11	33	44
		%	31.4 %	100.0 %	64.7 %
	Total	N	35	33	68
		%	100.0 %	100.0 %	100.0 %

*Abbreviations*: *CG* control group, *IG* intervention group

Data related to identification and experience with inclusion were also collected, prior to T1 (Table 2). For the independent variables (IV) gender, identification and experience with an inclusive school system no statistically significant relationship for the groups were found (Chi-Square Test).

**Table 2:** Distribution of experience and identification with inclusion

			CG	IG	Total
Experience Inclusion	No	N	17	14	31
		%	47.2 %	41.2 %	44.3 %
	Yes	N	18	19	37
		%	51.4 %	57.6 %	54.4 %
	Total	N	35	33	68
		%	100.0 %	100.0 %	100.0 %
Identification Inclusion	No	N	6	8	14
		%	16.7 %	23.5 %	20.0 %
	Yes	N	29	25	54
		%	82.9 %	75.8 %	79.4 %
	Total	N	35	33	68
		%	100.0 %	100.0 %	100.0 %

*Abbreviations*: *CG* control group, *EG* intervention group

### Statistical analyses

Using a two (group affiliation) × 2 (measurement time) between subjects design, the effects of the Acceptance and Commitment Training on the subjective tension of SEN students sample (*N* = 68) were examined. Furthermore, age and semester were used as covariates in order to control their influence on the results related to subjective tension levels. As possible indicators for maturity, it was assumed that age as well as semester could influence conflict behavior. Therefore the ANCOVA studied the effects of ACT on subjective tension of special education students, the effects of the training on the collaborative competence were examined using Chi-square test.

### ACT-Training

The ACT-Training course included 90-minute sessions, which were conducted for 12 fixed days. The first two sessions focused on the measurement of T1 and T2, respectively. The ten remaining dates were devoted to the Acceptance and Commitment Training. With an aim of intensifying the treatment, the intervention group was divided in three small groups, comprising 11 subjects each.

With regards to the content, the ACT implementation followed the recommendation of Flaxman and Bond [31], and Bond and Dryden [30]. However, slight modifications were carried out in order of the exercises (Table 3). During the ACT training, metaphors and experiential exercises were used to increase the effectiveness of psychological processes, which later led to the promotion of psychological flexibility and reduction of stress. The motives of ACT are elaborated below:

**Table 3:** ACT-Training

	Content	Exercises resp. metaphors
Stress and symptoms of stress	What does cause work stress in inclusive classrooms?	“Sink metaphor”
	What are your symptoms of stress?	
	The participants are told that the training focuses on changing how individuals react to stress. It is not about changing the sources of stress!	
		“Stress diary” (Homework)
“Creative hopelessness” and “control is the problem”	Beginning ACT:	“Do not think about …”
	What have you done to deal with stress and how that has worked for you? (Consequences of control)	“Quicksand-metaphor”
	Willingness as an alternative strategy	“Just noticing”(willingness exercise) (Homework)
Identify your stress buttons	Identify thoughts and emotions that cause stress	“How your mind works”(Exercise with adjectives)
	Get off your“buts”and replace“but”by the word“and”	“The I-cannot-Wall and the Gate-of-willingness”
		“Leaves on the stream”(Homework)
		“What to accept?”
Self as context	Do not struggle: find your observing-self	“Observer Exercise”(Homework)
	Think about the different roles you play in your live and which thoughts and feelings are linked to each role	“Sky and weather-metaphor”
		“Which roles are you playing in your live? ”
Defusion	Do not get caught up by thoughts, feelings, and emotions	“Thoughts as feathers”
		“This is just the role of… I am playing – naming the story”
Value clarification	What do you want your life to be about and what do you want to stand for	“Eulogy”
	To differ between values and goals	“Value diary”(Homework)
		“Value target”
Committed action	“There is nothing good: unless one does it!”	“Train metaphor”
	Goals, actions and barriers	“Operationalize your goals”
		“What could go wrong? ”
Committed action: NVC	Introducing the four-part Nonviolent Communication process	NVC-exercises for each part of the NVC-process [47]
	Practical testing of NVC during role plays with video feedback	Role plays

*Abbreviations*: *ACT* Acceptance and Commitment Training, *NVC* nonviolent communication

1. The training aims at promoting the acceptance of unpleasant thoughts, feelings and sensations, and thereby encourages the students to connect with the present moment (the mindfulness part).
2. The ACT-process enables a person to identify the difference between thoughts, feelings and reality, and thereby facilitates separation of an individual from unpleasant thoughts. This process is referred to as defusion. In the context of the study, students can defuse their thoughts and feelings and reduce experiential avoidance (the tendency to avoid unpleasant thoughts and feelings). Defusion can equip the students to move forward and undertake commitments that are in accordance with their personal values.

The exercises and metaphors used in the training (Table 3) can be found in Bond and Dryden [30] or in the book, titled, ‘Therapie-Tools: ACT’ [40]. The exercises included in this book are also available in the German language. Audio files for certain exercises were also provided to the participants with an aim of encouraging them to practice the exercises at home. The mindfulness exercises took place in groups, while individual value-related exercises were done in worksheets.

Subsequent to the discussions on committed action, during the training sessions, NVC was employed to provide skills training and theoretical input to the students [32]. The two seminars focused on linking values and goals to the basic principles of NVC. This principle focuses on equipping individuals to take committed action, which is guided by personal values. In the last part of the seminar, the communication skills of the students were tested through role plays. These role plays revealed muddled perceptions of students, which was cleared by compelling students to clarify their personal values. The students made video recordings of these role plays during the training. These video recordings might have helped students to assess their behavior during the role plays because they were able to improve their own occurrence and their effect on others.

## Results

While the data presentation for one part of the study is descriptive, certain parts of the study employed specific hypothesis tests to determine differences. Except for the subjective tension variable, the data were normally distributed. Therefore, hypothesis testing was based on nonparametric techniques. The data were analyzed using SPSS version 16 (SPSS Inc., Chicago, IL, USA). Only complete data sets were analyzed.

### Subjective tension

**Fig. 1:**
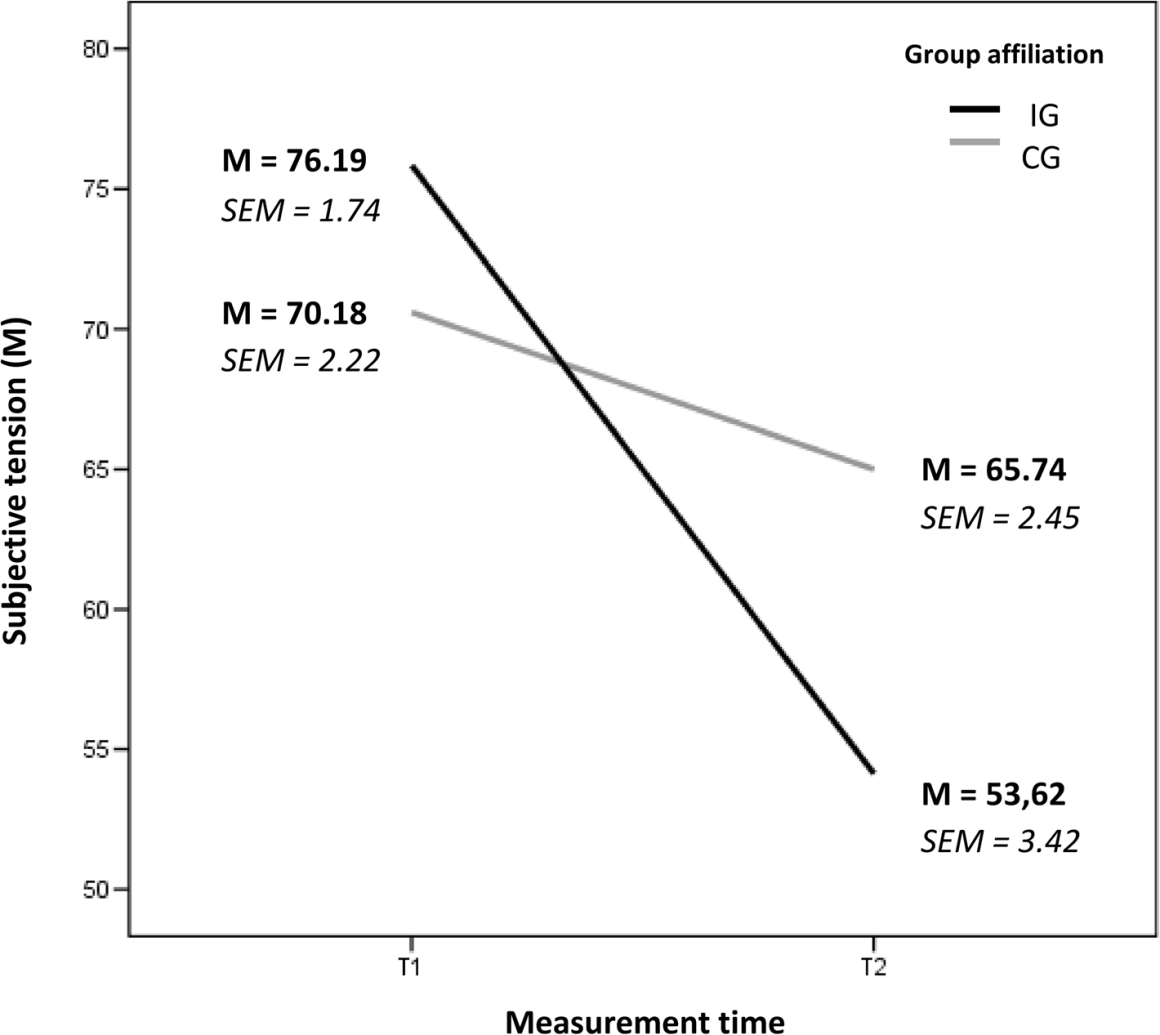
Subjective tension. Abbreviations: *CG* control group, *IG* intervention group, *T1* first time of measurement, *T2* second time of measurement, *M* mean; *SEM* standard error of mean

One of the principle results of this research focuses on subjective tension (Fig. 1). Subsequent to the role plays, all participants were asked to rate their subjective tension on a scale from 0 to 100 (‘not tensed’ to ‘fully tensed’).

The mean value for tension for the IG *M* = 76.19 (SD = 9.84; SEM = 1.74) at T1 was above the average of the CG with a mean value of *M* = 70.18 (SD = 12.93; SEM = 2.22), but no significant differences between groups could be found. A decrease of tension over time was demonstrated by the IG with a mean value of *M* = 53.62, (SD = 19.35; SEM = 3.42) at T2, although the standard deviation increased. The mean value for the CG showed only a slight decrease to *M* = 65.74, (SD = 14.31; SEM = 2.45). An analysis of covariance (ANCOVA) was conducted (Table 4) and a significant interaction for group affiliation and measurement time was found (F_(1,64)_ = 6.821 → *p* = 0.011; η^2^p = 0.099). Since in this case (one repeated measurement) sphericity can not be violated, no corrections for violations of sphericity is necessary. The effects of the variables, age and number of semester, were statistically controlled, but both the variables did not have any significant effect (Table 5).

**Table 4:** ANCOVA (within-subjects effects)

Type III Sum of Squares	Df	Mean Square	F	Sig.	Type III Sum of Squares
MT	67.740	1	67.740	.428	.515
MT * Age	12.617	1	12.617	.080	.779
MT * Semester	3.595	1	3.595	.023	.881
MT * Groupaffiliation	1078.566	1	1078.566	6.821	.011
Error (MT)	9804.366	62	158.135		

*Abbreviations*: *df* degrees of freedom, *F* observed F value, *Sig* significance, *MT* measurement time

**Table 5:** ANCOVA (between-subjects effects)

Source	Type III Sum of Squares	df	Mean Square	F	Sig.
Intercept	19232.554	1	19232.554	77.696	.000
Age	753.622	1	753.622	3.044	.086
Semester	895.756	1	895.756	3.619	.062
Group-affiliation	232.877	1	232.877	.941	.336
Error	15347.286	62	247.537		

*Abbreviations*: *df* degrees of freedom, *F* observed F value, *Sig* significance

### Conflict handling modes

**Fig. 2:**
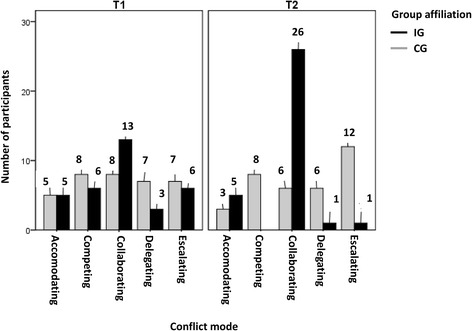
Assessment of conflict behavior (frequencies). Abbreviations: *CG* control group, *IG* intervention group, *T1* first time of measurement, *T2* second time of measurement

Subsequent to the completion of role plays, the conflict behavior of each participant was assigned to the conflict handling modes. The number of participants of the CG assigned to the conflict handling mode, ‘accommodating,’ at T1 decreased over time, from 5 to 3 (Fig. 2). The conflict handling modes ‘collaborating’ and ‘delegating’ also showed a marginal decline in the CG. During the second phase of assessment (T2), these participants chose the conflict handling mode, ‘escalating,’ which increased up to 12. The number of individuals who competed and pursued their own interests did not change.

However, a different picture emerged for the IG. The number of participants with conflict handling mode, ‘accommodating’ stayed the same, and the conflict handling mode, ‘competing’ disappeared completely. At T2, only one participant was remaining, who wanted to delegate or escalate the conflict to a third party—principal. The number of participants showing the conflict handling mode, ‘collaborating’ doubled.

During the second phase of assessment (T2), conducted using the Chi-square test, the group differences (*χ*^2^ = 10.59; *p* = 0.00) and the changing over time for the IG (*χ*^2^ = 25.91; *p* = 0.00) presented significant results. With an aim of meeting the requirements for the Chi-square test, it was necessary to combine cells and obtain an expected frequency of more than five for each cell [41]. Having established the fact that collaborative conflict handling mode enhances professional relationship and pursues the interests of the conflicting parties, it was deemed as the only criterion for successful conflict solving. The remaining conflict handling modes were combined in one cell for the Chi-square test, as these modes did not contribute towards stabilizing or enhancing the relationship.

A high inter-rater reliability, established through Krippendorff’s alpha of .83, supported the validity of the data [42] (Table 6). Krippendorff’s alpha evaluates the agreement of independent raters.

**Table 6:** Krippendorff’s Alpha

	Alpha	LL95 % CI	UL95 % CI	Units	Observer	Pairs
Nominal	0.8289	0.7604	0.8973	10	6	150

*Abbreviations*: *LL95 % CI* lower limit of the 95 % confidence interval, *UL95 % CI* upper limit of the 95 % confidence interval

### Results concerning the acceptance of the training

The participants were asked to evaluate the ACT training, after the completion of the training (Table 7). The feedback revealed that all the participants emphasized on the practical relevance of ACT. The need of two thirds of the participants was addressed by the contents of the training, and the results convinced these participants to recommend the course to other teachers. As the ACT training drew to close, 54.8 % of participants expressed a desire to continue with ACT and 61.3 % were looking forward to apply the learning from the training to everyday life.

**Table 7:** Assessment of the ACT-Training

	*N*	%
Feel addressed (*N* = 32)		
Yes	24	75.0
No	8	25.0
Practical relevance (*N* = 33)		
Yes	33	100.0
No	0	0.0
Recommendation (*N* = 33)		
Yes	22	66.7
No	11	33.3
Willing to continue (*N* = 31)		
Yes	17	54.8
No	14	45.2
Transfer (*N* = 31)		
Yes	19	61.3
No	12	38.7

*Abbreviations*: *N* number of participants

Lastly, it can be stated that the subjective tension of the IG as well as their conflict handling behavior had changed fundamentally over the training period, within the context of the hypothesis. Furthermore it can be noted that the training was highly accepted by the students.

## Discussion

The current study examined the effectiveness of Acceptance and Commitment Training (ACT) in reducing stress and enhancing collaboration among SEN students. The authors set up two hypotheses to establish that psychoeducational training (ACT) linked with practical experience can increase collaborative competence. The hypotheses test ACT’s effectiveness in reducing subjective tension (H1) and equipping SEN students with an effective and successful conflict handling mode—collaboration (H2).

### Subjective tension

The decrease in subjective tension, recorded from the first to second assessment (Fig. 1), formed one of the two main hypotheses of the authors. The authors based their assumption on the premises that values clarification and willingness would lead SEN students to take committed action, according to personal values. Besides, the authors also proposed the implementation of ACT for successful conflict handling. At the backdrop of these assumptions, students with an average age of 27 and limited practical experience were considered for the study. As per assumptions, a lack of well-rounded professional and personal development is generally evident among students of the selected age group. Considering this need for well-rounded development, the motive of ACT would be to assist participants in the development of a stable value system. These values impart an occupational identity to the students, which continue to guide them in inclusive class settings and reduce subjective tension.

The aforementioned data confirm this reduction in H1 (subjective tension) (Fig. 1). The intervention design with IG supports the assumption that the ACT training is responsible for the significant decrease of subjective tension for IG. However, a similar decrease was not found in the CG, as the participants could anticipate the course of conversation (in the sense of a certain habituation) during role plays. The results for the variables, age and number of semesters, in the conducted analysis of covariance were not statistically significant (Table 5).

### Conflict handling modes

As stated earlier, a more successful form of conflict resolution forms the second main hypothesis. Successful conflict handling can only be found in a collaborative conflict handling mode, which pursues the interests of both conflict parties. Therefore, it is considered to be the most favorable medium to enhance professional relationship. Collaboration requires a party to put in a lot of mental and emotional effort to understand the reasons behind the behavior of its counterpart. According to their value systems, students tried to communicate in a compassionate and non-violent way to enhance the quality of connection between the interlocutors. In this context, it can be assumed that students would only undertake collaborative efforts, if they are really convinced about collaboration being an effective conflict handling mode. If survival in role plays would be the primary goal of participants, it can be stated that ‘delegating’ or ‘escalating’ and ‘accommodating’ involve lesser mental efforts than ‘collaborating,’ and therefore could be more easily and economically employed to achieve conflict resolution, especially on the short run.

The standards of communication in the collaborative conflict handling mode are very high, and these demands have a notable impact on participants in the CG. As shown in Fig. 2, the conflict handling mode of 18 participants in the CG changed to delegating or escalating mode. It can be assumed that the ‘reality shock,’ resulting from the communication requisites of the ‘collaborative’ mode, compelled the students without the training to reconsider ‘delegating’ or ‘escalating’ as the only possible conflict handling mode.

Figure 2 also shows that the conflict handling mode of the IG completely changed from T1 to T2. A total of 26 out of 33 participants were willing to collaborate. The group of people with the conflict handling mode ‘accommodating’ stayed the same. It is interesting to note that few participants could not relate to the inclusive education framework. These remaining participants delegated or escalated the conflict to a third party – the principal.

The results indicate that participants with the collaborative conflict handling mode could successfully resolve conflicts. Therefore, it can be inferred that ACT leads the participants to handle conflicts successfully, as it is assumed in H2.

### Results concerning the acceptance of the training

Developed along the premises of ACT, the aim of the study was to develop and evaluate an education module, which would prepare SEN students for communication, interaction and problem solving in inclusive settings. The need for this kind of module arose to reduce stress that could have possibly accompanied the recent educational reforms. The significance of ACT-training was highlighted by the fact that two-thirds of the participants were benefitted by the content of the training, and these students were planning to recommend the training to fellow students. Having reaped the benefits of ACT, 54.8 % expressed a desire to continue with the ACT-training in future.

Therefore, it is emphasized that participants agree to the practical relevance of the ACT-training (Table 7). The acceptance of participants would indicate their awareness regarding the importance and complexity of collaboration in inclusive settings.

### Limitations

Although the study benefitted most of its participants, it also revealed distinct weaknesses. The ACT-training was part of the Masters curriculum, and hence it was not justified to randomly assign students to IG and CG. The students in IG were part of the Masters course, whereas most of the CG members pursued the Bachelor’s program. As master’s students possessed advanced knowledge it could be assumed that master’s students were able to apply their experience at T2 in a better manner than the participants of the CG. This assumption is supported by the findings at T1, which reveal that 13 out of 33 participants in IG sought for collaboration, before commencement of the training (Fig. 2).

The ANCOVA conducted for controlling variables, age and number of semester, did not reveal significant effects. The results for these two variables were not significant, and additional studies involving more number of subjects were needed to determine the impact of these variables on the decrease of subjective tension.

The difference in subjective tension between both groups is another limitation that has been highlighted since the beginning of the study. The difference between both the groups is not statistically significant. However, the difference is recorded to be around half the size of the standard deviation. While indicating a potential sampling bias, this outcome points toward stress levels associated with the Masters course. Therefore, additional studies are needed to evaluate a potential bias in the sampling, and provide valid reasons behind the recorded difference in subjective tension between both groups.

In addition, the analysis did not account for the fact that students received intervention in three different groups, which may have introduced further covariance among participants.

## Conclusions

The data presented show that an Acceptance and Commitment Training enables participants to solve conflicts successfully, and simultaneously reduces subjective tension. Being forced to clarify ones values might change the interpretation of students about collaboration and provides orientation for self-confident action. Considering the fact that students have the skills to interact in unfamiliar situations with other teachers, committed action in accordance with personal values can additionally be encouraged by practicing NVC.

It is worth noticing that SEN students sometimes are unwilling to work in inclusive settings (about 20 % in the present study). These students do not support the idea of inclusion. However, some schools do not show any consideration toward this fact, and students will be forced to work in inclusive classrooms. The compulsion to work in an inclusive setting, without having the choice to weigh the advantages and disadvantages of collaboration, might put teachers under great pressure. In addition, the possibilities of structuring procedures of an inclusive classroom independently might cause a surge in the stress levels of teachers. It is important to devote time and further a normative discourse to implement such reforms effectively. It takes a lot of time to adapt to being a team player and to internalize the new set of values that accompany the inclusive framework. There is a need for a common goal, and a need to identify this common goal. An ACT-training could contribute towards building a new professional identity, on the basis of clarified personal values, which would drive the teacher look for common goals. This new professional identity could also lead to revaluation of potential stressors, often found in inclusive classrooms. Therefore, as shown in the study, the identification of possible stressors might either facilitate stress reduction or lead the concerned teacher to find alternative career options.

The present study also indicates that even the special education students, who should be prepared for working in inclusive settings, are still underprepared. There are postulations of the Standing Conference, especially the demands to prepare students for ‘communication, interaction and conflict handling’ [38], but initiatives to realize these objectives have not been grasped within the wide-reaching reforms of the academic structure in teacher education, at least concerning the native state of Schleswig-Holstein. A shift from establishing professional skills toward establishing professional and personal skills should be the logical consequence of ACT-training − stress reduction and promoting collaboration. Therefore, the Federal States must base their propositions for structuring collaboration on best practice experiences.

Further research should investigate the effectiveness of ACT-training to reduce stress of teachers facing the change into an inclusive school system.

Besides, from a biological perspective, emphasis should be given on the evaluation of stress systems through non-invasive biomarkers (e.g., cortisol or alpha-amylase). At the moment, there have been rare attempts to look for these non-invasive biomarkers (e.g. [43–46]), and such biological evaluations with respect to inclusive settings have been disregarded. However, one study supports the notion of altered HPA axis regulation in chronically work-stressed teachers [46], and the results for subjective tension in the current study might confirm and complement these findings for inclusive school settings. Therefore, longitudinal studies in these settings are necessary and the results of this study should offer sufficient starting points for psychoneuroendocrinological research.

An Acceptance and Commitment Training can be seen as an appropriate additional measure for initiating effective collaboration and stress alleviation. Therefore, it can be concluded that ACT is an appropriate and practical technique to maintain the health of teachers.

## Availability of supporting data

Supporting data is available upon request form the corresponding author.
